# A microencapsulated feed additive containing organic acids and botanicals has a distinct effect on proliferative and metabolic related signaling in the jejunum and ileum of broiler chickens

**DOI:** 10.3389/fphys.2023.1147483

**Published:** 2023-03-22

**Authors:** Casey N. Johnson, Ryan J. Arsenault, Andrea Piva, Ester Grilli, Christina L. Swaggerty

**Affiliations:** ^1^ Southern Plains Agricultural Research Center, Agricultural Research Service, United States Department of Agriculture, College Station, TX, United States; ^2^ Department of Animal and Food Sciences, University of Delaware, Newark, DE, United States; ^3^ DIMEVET, University of Bologna, Bologna, Italy; ^4^ Vetagro S.p.A, Reggio Emilia, Italy; ^5^ Vetagro Inc., Chicago, IL, United States

**Keywords:** broiler chicken, antibiotic alternatives, botanicals (thymol and vanillin), essential oils, organic acids (citric and sorbic), immunometabolism, kinome, gut health

## Abstract

Well designed and formulated natural feed additives have the potential to provide many of the growth promoting and disease mitigating characteristics of in-feed antibiotics, particularly feed additives that elicit their effects on targeted areas of the gut. Here, we describe the mechanism of action of a microencapsulated feed additive containing organic acids and botanicals (AviPlus^®^P) on the jejunum and ileum of 15-day-old broiler-type chickens. Day-of-hatch chicks were provided *ad libitum* access to feed containing either 0 or 500 g/MT of the feed additive for the duration of the study. Fifteen days post-hatch, birds were humanely euthanized and necropsied. Jejunum and ileum tissue samples were collected and either flash frozen or stored in RNA-later as appropriate for downstream applications. Chicken-specific kinome peptide array analysis was conducted on the jejunum and ileum tissues, comparing the tissues from the treated birds to those from their respective controls. Detailed analysis of peptides representing individual kinase target sites revealed that in the ileum there was a broad increase in the signal transduction pathways centering on activation of HIF-1α, AMPK, mTOR, PI3K-Akt and NFκB. These signaling responses were largely decreased in the jejunum relative to control birds. Gene expression analysis agrees with the kinome data showing strong immune gene expression in the ileum and reduced expression in the jejunum. The microencapsulated blend of organic acids and botanicals elicit a more anti-inflammatory phenotype and reduced signaling in the jejunum while resulting in enhanced immunometabolic responses in the ileum.

## 1 Introduction

Natural products are being explored as potential antibiotic alternatives for use in animal agriculture. Promising alternatives include, but are not limited to, organic acids, botanicals, short chain fatty acids, and bacterial fermentates (Postbiotics) (Johnson et al., 2019; Swaggerty et al., 2020a; 2020b; Liu et al., 2021; Salminen et al., 2021). These natural products have been used and evaluated in the animal agriculture industry for a number of years and numerous primary and review articles in the literature support the use of such products for use as antibiotic alternatives, to enhance production parameters, and improve animal health and welfare (Zeng, et al., 2015; Abd El-Hack et al., 2016; Jazi et al., 2018; Yadav and Jha, 2019). Extensive studies are being conducted to measure and evaluate the performance enhancing and disease mitigating effects of these products (EFSA Panel et al., 2020 on Additives and Products or Substances used in Animal Feed (FEEDAP); Swaggerty et al., 2020a; 2020b; 2022). Microencapsulated feed additives have been shown to allow such products to survive the upper gastrointestinal tract (GI tract) and be delivered along the small intestine (Grilli et al., 2007; Piva et al., 2007). The small intestine is the main site of digestion and nutrient absorption in chickens (Svihus, 2014). The different gut segments have been reported to serve different functions in relation to nutrient absorption, with the jejunum being the major site of absorption of fat, starch, and protein, and the ileum, anatomically following the jejunum, being the major site of water and mineral absorption (Svihus, 2014). Although microencapsulation allows for the product to be delivered to the small intestine, the concentration of included compounds have been shown to decrease down the length of the gut (Grilli et al., 2007; Piva et al., 2007).

The chicken-specific kinome peptide array and accompanying software are designed to measure and evaluate the immunometabolic signaling changes that occur between treatment and control groups (Jalal et al., 2009; Li et al., 2012). The technology utilizes 15 amino acid (AA) long peptides from the chicken proteome corresponding to known kinase target sites in the human proteome (orthologues sequences). Kinases are enzymes which catalyze phosphorylation events, the transfer of a phosphate group from ATP to a target protein, and act as key regulators of cell signaling. This post-translational modification can act to increase or inhibit the target protein’s activity and modulate its capacity to interact with other molecules. Proteins also can contain multiple kinase target sites sometimes with complementary or enhancing functions and sometimes with antagonistic or competing functions. In the peptide array assay, active kinases in the samples phosphorylate their target sites represented on the array. Many sites on the array have known physiological functions in the human phospho-proteome and therefore can provide valuable insight into chicken intracellular signaling cascades and thus physiological changes between treatment groups (Jalal et al., 2009). Using a kinomic approach to explore the changes to intracellular signaling cascades associated with immune and metabolic processes in response to the blend, we are able to better understand the changes conferred resulting in improved production measures and disease outcomes (Swaggerty et al., 2020a; Swaggerty et al., 2022).

There is a well-documented complex interplay between cellular metabolism and immunity and *vice versa*, the study of which is deemed immunometabolism (Pearce and Pearce, 2013). The emphasis on immunometabolic signaling allows researchers to look closely at the immune and metabolic changes imparted by products, therapeutics, and diseases in tissues (*in vivo*), cell populations (*ex vivo*), or monocultures (*in vitro*). Using the immunometabolic chicken-specific kinome peptide array we are able to elucidate differences in key cell signaling cascades which can provide insight into the immune and metabolic alterations imparted under different treatment conditions. Thus, providing insight into potential mode-of-action.

In a previous study, our group determined the kinome profile of jejunum and ileum tissue samples from birds fed the microencapsulated blend of organic acids and botanicals compared to corresponding tissue samples from control fed birds. That analysis provided the framework for the present study, providing valuable insight into the changes in cell signaling pathways conferred by the product and highlighting the presence of a distinction between the tissue responses (Swaggerty et al., 2020a). The objectives of the current study were to further characterize the mechanism of action of this microencapsulated blend of organic acids and botanicals by defining the site-specific changes in phosphorylation status of proteins and inferring the physiological consequences of those changes. Further elucidating the mode of action of these products will allow us to create more efficacious health promoting products that will help mitigate foodborne and poultry diseases while increasing production efficiency.

## 2 Materials and methods

### 2.1 Experimental design and chickens

Trial was conducted as previously reported (Swaggerty et al., 2020a) and briefly summarized here. Day-of-hatch by-product broiler breeder chicks (from here on referred to simply as broilers) were obtained from a commercial hatchery (Timpson, TX). Chicks were placed in 3 m × 3 m floor pens containing pine shavings and provided *ad libitum* access to feed and water throughout the study. Chicks were fed a corn and soybean meal starter diet that met or exceeded the established nutrient requirements of poultry (National Research Council, 1994). Control birds were fed a non-supplemented diet while treatment birds were fed the same diet base supplemented with 500 g/metric ton (MT) of a commercially available lipid-microencapsulated blend of citric (25%) and sorbic (16.7%) acids, thymol (1.7%), and vanillin (1.0%) (AviPlus®*p*, Vetagro S. p.A, Reggio Emilia, Italy). Two replicate trials were conducted. From each replicate trial control (n = 10) and treatment (n = 10) birds were euthanized by cervical dislocation and necropsied at 15 days post hatch. Sampling time was established based on previous work (Grilli et al., 2015; Swaggerty et al., 2020a) and in consideration of the productive cycle of broilers. In a commercial setting, the first diet change typically occurs between 10 and 15 days-of-age. Additionally, the first 2 weeks of grow out are critical to the development of the gastrointestinal and immunological function and by 2–3 weeks of age broilers are considered mature. All bird studies were overseen by the on-site attending veterinarian under the approved experimental procedures outlined in protocol #2017008 and were approved by the USDA/ARS Institutional Animal Care and Use Committee. The IACUC operates under the Animal and Plant Health Inspection Service (APHIS) establishment number 334299. The experiments were conducted in accordance with the recommended code of practice for the care and handling of poultry and followed the ethical principles according to the Guide for the Care and Use of Agricultural Animals in Research and Teaching, fourth edition (Ag Guide, 2020).

During necropsy, jejunum and ileum samples were taken and stored for later use. Samples for the kinome peptide array protocol were flash frozen in liquid nitrogen and stored at -80°C. Samples for quantitative real-time RT-PCR (qRT-PCR) were placed in RNA-Later (Qiagen, Valencia, CA) and stored at -20°C for later use.

### 2.2 Chicken-specific immunometabolic kinome peptide array

Kinome peptide array protocol was carried out as previously described (Arsenault et al., 2017) and briefly summarized here. Samples from 5 birds per replicate trial (n = 10) were used to represent each treatment group, the same birds were used for both tissues (jejunum and ileum). Tissue samples (40 mg) were submerged in PBS and vortexed to clear the sample of any gut contents and homogenized by a Bead Ruptor 24 homogenizer (Omni International, Kennesaw, GA) in 100 μL of lysis buffer containing protease inhibitors. An activation mix containing ATP was then added to the lysate and applied to peptide arrays. Peptide arrays have 771 15-mer peptides each printed 9 times per array. These peptides represent known kinase target sites. Kinases present in our samples are able to phosphorylate target peptides using the ATP supplied in the activation mix. Arrays were then incubated at 37°C with 5% CO2 in a humidity chamber, allowing kinases in the samples to phosphorylate their target sites. Samples were then washed and a phospho-specific florescent stain was applied. Stain not bound to phosphorylated sites was removed *via* a de-staining process. Arrays were then imaged using a Tecan PowerScanner microarray scanner (Tecan Systems, San Jose, CA) at 532–560 nm with a 580-nm filter to detect dye fluorescence.

GenePix Pro software (Molecular Devices, San Jose, CA) was then used to grid the array images, ensuring peptide spots were correctly associated with their phosphorylation site and the spot intensity signal was collected. Greater intensity florescence correlates to greater phosphorylation at the target site. Florescent intensities for treatments were then compared with those for controls using the data normalization and analysis program, Platform for Intelligent, Integrated Kinome Analysis 2 (PIIKA 2) (Li et al., 2012; Trost et al., 2013). PIIKA two performs data normalization and variance stabilization prior to making specified comparisons to generate fold-change values (treatment/control) and significance values (*p*-value). *p*-values are calculated *via* one-sided paired *t*-test between treatment and control for a given peptide. The resulting data output was then used in downstream applications such as Search Tool for the Retrieval of Interacting Genes/Proteins (STRING) (Szklarczyk et al., 2021) and Kyoto Encyclopedia of Genes and Genomes (KEGG) (Kanehisa et al., 2021) databases used to pinpoint changes in protein-protein interactions and signal transduction pathways.

### 2.3 RNA extraction for quantitative real-time reverse transcription polymerase chain reaction

Jejunum and ileum tissue samples (30–40 mg) were placed in BeadBugTM tubes (#Z763799-50 EA, Sigma-Aldrich^®^, St. Lois, MO). Lysis buffer (600 μL; RNeasy Mini Kit; Qiagen) was added and the samples were homogenized in the Omni International Bead Ruptor Elite (Kennesaw, GA) on speed setting six for 2 min. Total RNA was then isolated from the homogenized samples according to the manufacturer’s instructions, eluted with 50 μL of RNase-free water, and stored at -80°C until quantitative real-time reverse transcription polymerase chain reaction (qRT-PCR) analyses were performed.

### 2.4 Quantitative real-time RT-PCR for mRNA expression

mRNA expression was analyzed for Interferon gamma (IFNγ), Interleukin 4 (IL-4), and Hypoxia Inducible Factor 1 alpha (HIF-1α) in jejunum and ileum. All primer and probe sequences are provided in Table 1. Genes of interest were selected due to up and downstream relation to pathways of interest identified during the kinome peptide array data analysis. The qRT-PCR was performed using the TaqMan one-step RT-PCR master mix reagents (Applied Biosystems, Branchburg, NJ). Amplification and detection of specific products were performed using the Applied Biosystems 7,500 Fast Real-Time PCR System with the following cycle profile: one cycle of 48°C for 30°min and 95°C for 20°s and 40 cycles of 95°C for 3°s and 60°C for 30 s. Quantification was based on the increased fluorescence detected by the 7,500 Fast Sequence Detection System due to hydrolysis of the target-specific probes by the 5′ nuclease activity of the rTth DNA polymerase during PCR amplification. Sample standardization was done using 28 S RNA. Results were calculated as 40-cycle threshold (CT) for each tissue sample from control- and supplement-fed chickens and the data are presented as the fold-change from controls. Fold change was calculated as 2^(supplement-fed corrected mean–control-fed corrected mean) for each tissue.

**TABLE 1:** Primer and probe sequences for qRT PCR.

RNA target	Forward (5′ to 3′)	Reverse (5′ to 3′)	Probe (5′ to 3′)	References	Accession #
28s	GGC GAA GCC AGA GGA AAC T	GAC CGA TTT GCA CGT C	6FAM-AGG ACC GCT ACG GAC CTC CAC CA-TAMRA	Kaiser et al. (2000)	X59,733
IFNy	GTG AAG GTG AAA GAT ATC ATG GA	GCT TTG CGC TGG ATT CTC A	6FAM-TGG CCA AGC TCC CGA TGA ACG A-TAMRA	Kaiser et al. (2000)	Y07922
IL-4	AAC ATG CGT CAG CTC CTG AAT	TCT GCT AGG AAC TTC TCC ATT GAA	6FAM-AGC AGC ACC TCC CTC AAG GCA CC-TAMRA	Avery et al. (2004)	AJ621735
HIF-1α	GCA GAC TCA GAC ACC ATC AA	GGA CAG GAG ATG GAA CAA	6FAM-TGG CAG AAC GTG TGG ATG TGA -TAMRA	Present Study	NM_204,297

## 3 Results

### 3.1 Kinome analysis

#### 3.1.1 Protein and peptide assessment

**FIGURE 1:**
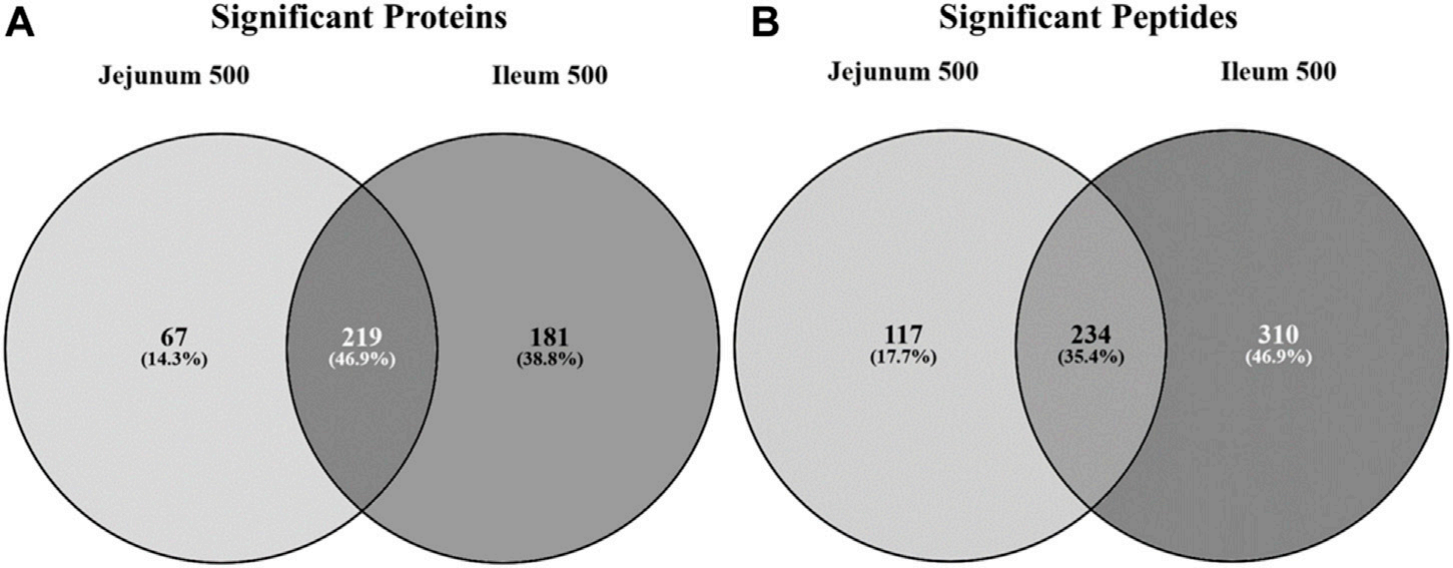
Venn diagram of significantly differentially phosphorylated proteins and peptides in ileum and jejunum from birds provided feed supplemented with 500 g/MT of a microencapsulated blend of organic acids and botanicals compared to their respective controls fed a non-supplemented diet at 15 days post-hatch. Kinase target peptides are printed on the array and are phosphorylated by active kinases; there are multiple peptides present on the array for a given protein. Proteins **(A)** and peptides **(B)** statistically significantly differentially phosphorylated in jejunum and ileum from birds fed product containing feed (*p* < 0.05) from their respective control tissue were input into the Venn diagram-generating program Venny (http://bioinfogp.cnb.csic.es/tools/venny/).

Statistically significant differentially phosphorylated peptides were identified by kinome peptide array analysis in ileum and jejunum tissue samples collected. The Venn diagrams shown in Figure 1 highlight the differential treatment effect between the two tissues. The overlapping and distinct alterations in phosphorylation events on proteins represented on the array are shown in Figure 1A while the distinct and overlapping peptides represented on the array with significantly altered phosphorylation status are shown in Figure 1B. Using both Venn diagrams, the distinction between significant changes to individual protein phosphorylation status and changes to different peptides on those proteins can begin to be observed.

Figure 1A shows that the largest proportion of proteins with statistically significant differential phosphorylation events between treatment and control tissues (219 proteins) are shared between the two tissues (46.9%). While a large proportion (35.4%) of the peptides significantly differentially phosphorylated are indeed overlapping between the two tissues (234 peptides) (Figure 1B), the largest proportion (46.9%) are significantly differentially phosphorylated in the ileal tissue (310 peptides). The remaining 17.7% of significantly differentially phosphorylated peptides are in the jejunum (117 peptides).

**FIGURE 2:**
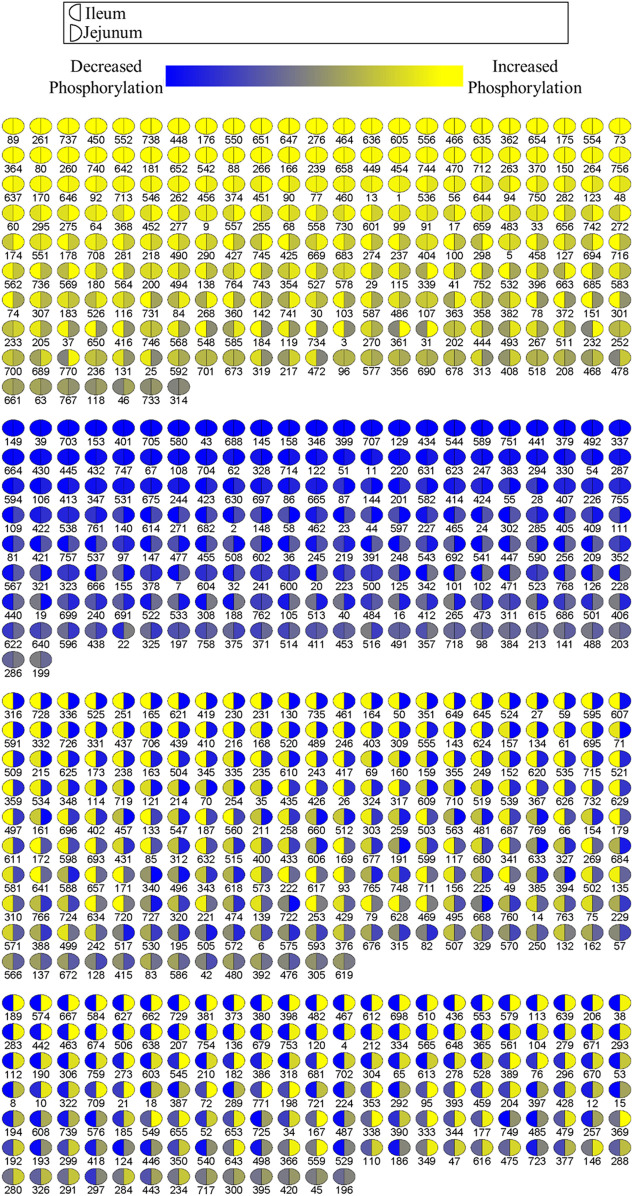
Comparison of relative phosphorylation of array peptides between ileum and jejunum due to product. Each spot represents a peptide on the array which corresponds to a kinase recognition site. The left half of each spot shows the differential phosphorylation status relative to control birds for ileum. The right side of each spot shows the phosphorylation status relative to control birds for jejunum.

A visualization comparison of the kinome results from the ileum and jejunum was generated by PIIKA two showing the phosphorylation level of each spot compared to control (Figure 2) (Trost et al., 2013). Ileum phosphorylation level is represented on the left side of each circle while jejunum phosphorylation level is on the right side. Figure 2 shows that ∼52% of the peptides on the array share a common phosphorylation status in both ileum and jejunum (or is not statistically significant). The other ∼48% of the peptides show a differential phosphorylation state between jejunum and ileum; i.e. increased in one and decreased in the other (or is not statistically significant). This relatively large proportion of peptides that display opposite phosphorylation status between the two tissues indicates distinct responses to the product and/or an effect of delivery of the product down the GI tract.

Collectively, the Venn diagrams show that even though the same proteins are affected in the two tissues they are likely to have different peptides being differentially phosphorylated between the tissues. Additionally, the peptides shared between the tissues in Figure 1B may be differentially phosphorylated in opposite directions, as indicated in Figure 2. Taken together, these figures show that the treatment has a distinct effect between the two tissues, including differential effects on the same proteins.

#### 3.1.2 KEGG and reactome pathways

The proteins significantly altered for each tissue (relative to control) were input into the STRING protein-protein interaction database to generate a list of KEGG pathways overrepresented in the data (Szklarczyk et al., 2021) highlighting the physiology and response to stimuli within a specific tissue. The top 30 overrepresented KEGG pathways for each tissue are shown in Table 2. Many of the top pathways in both tissues are broadly related to growth and proliferation (Mitogen-activated protein kinase (MAPK), cancer, phosphatidylinositol 3-kinase- RAC (Rho family)-alpha serine/threonine-protein kinase (PI3k-Akt)) and specifically glucose metabolism (insulin, central carbon metabolism in cancer, Hypoxia-Inducible Factor-1 (HIF-1) signaling). As these pathways represent a physiological response centered around growth/proliferation and glucose metabolism and, as we have reported previously (Perry et al., 2020), glycolysis is closely related to inflammation and innate immunity, these pathways were considered in more detail.

**TABLE 2:** Top 30 KEGG Pathways Enriched in Jejunum and Ileum. The top 30 KEGG pathways, as determined by STRING, for each tissue are represented here. Proteins showing statistically significant differential phosphorylation status between treatment and control fed birds for each tissue were provided to STRING for protein-protein interaction analysis. False Discovery Rate (fdr) is a significance metric (*p*-value) provided by STRING. *p*-values are generated using the Benjamini–Hochberg procedure. Key pathways in immune and metabolic regulation have been bolded.

Top 30 KEGG pathways in jejunum		Top 30 KEGG pathways in ileum	
KEGG pathway	fdr	KEGG pathway	fdr
**MAPK signaling pathway**	**4.93E-39**	Pathways in cancer	6.03E-41
**PI3K-Akt signaling pathway**	**2.32E-32**	**MAPK signaling pathway**	**2.66E-36**
Pathways in cancer	2.32E-32	MicroRNAs in cancer	1.64E-32
**Insulin signaling pathway**	**1.90E-29**	**PI3K-Akt signaling pathway**	**1.81E-32**
Ras signaling pathway	3.25E-29	**Insulin signaling pathway**	**4.70E-31**
ErbB signaling pathway	1.12E-28	Hepatitis B	1.84E-27
MicroRNAs in cancer	1.68E-28	Proteoglycans in cancer	1.53E-26
Neurotrophin signaling pathway	1.28E-26	**HIF-1 signaling pathway**	**2.83E-26**
Hepatitis B	3.58E-25	**Central carbon metabolism in cancer**	**2.83E-26**
**Central carbon metabolism in cancer**	**5.20E-24**	ErbB signaling pathway	1.21E-25
Growth hormone synthesis, secretion and action	6.37E-24	Shigellosis	3.87E-25
PD-L1 expression and PD-1 checkpoint pathway in cancer	3.58E-23	**AMPK signaling pathway**	**5.89E-25**
EGFR tyrosine kinase inhibitor resistance	4.94E-23	Neurotrophin signaling pathway	1.70E-24
Focal adhesion	1.09E-22	Kaposi sarcoma-associated herpesvirus infection	1.70E-24
T cell receptor signaling pathway	5.70E-22	Human cytomegalovirus infection	2.64E-24
Chemokine signaling pathway	2.43E-21	EGFR tyrosine kinase inhibitor resistance	4.04E-24
Proteoglycans in cancer	1.01E-19	Ras signaling pathway	7.86E-24
Acute myeloid leukemia	2.19E-19	*Yersinia* infection	1.47E-23
**HIF-1 signaling pathway**	**3.06E-19**	T cell receptor signaling pathway	1.13E-22
Kaposi sarcoma-associated herpesvirus infection	3.06E-19	FoxO signaling pathway	2.37E-22
Insulin resistance	3.39E-19	Prostate cancer	3.94E-22
Prostate cancer	5.73E-19	Insulin resistance	3.98E-22
FoxO signaling pathway	7.68E-19	Hepatitis C	4.62E-22
Human cytomegalovirus infection	1.08E-18	PD-L1 expression and PD-1 checkpoint pathway in cancer	6.19E-22
Autophagy - animal	1.16E-18	Focal adhesion	4.49E-21
**mTOR signaling pathway**	**2.28E-18**	Chemokine signaling pathway	6.24E-21
Prolactin signaling pathway	5.79E-18	Osteoclast differentiation	8.12E-21
Glioma	1.13E-17	Glucagon signaling pathway	1.22E-20
Rap1 signaling pathway	1.36E-17	Growth hormone synthesis, secretion and action	3.78E-20
B cell receptor signaling pathway	4.03E-17	Rap1 signaling pathway	5.80E-20

In addition to KEGG pathway analysis, Reactome pathways were also determined. The top 30 Reactome pathways overrepresented in the data for jejunum and ileum are shown in Table 3. Similarly, many of the Reactome pathways also indicate alterations in growth and metabolic related signaling, but also highlights the immune related signaling changes. Of note are the pathways related to Toll-like receptor (TLR) signaling and those that indicate changes in Nuclear factor kappa B (NFκB) signaling, which will be further evaluated herein.

**TABLE 3:** Top 30 Reactome Pathways Enriched in Jejunum and Ileum. The top 30 Reactome pathways, as determined by STRING, for each tissue are represented here. Proteins showing statistically significant differential phosphorylation status between treatment and control fed birds for each tissue were provided to STRING for protein-protein interaction analysis. False Discovery Rate (fdr) is a significance metric (*p*-value) provided by STRING. *p*-values are generated using the Benjamini–Hochberg procedure. Key pathways in immune and metabolic regulation have been bolded.

Top 30 reactome pathways in jejunum		Top 30 reactome pathways in ileum	
Reactome pathway	fdr	Reactome pathway	fdr
Signaling by Receptor Tyrosine Kinases	6.23E-38	**Immune System**	5.27E-42
Signal Transduction	6.25E-36	Signal Transduction	9.23E-40
**Immune System**	**3.25E-35**	Signaling by Receptor Tyrosine Kinases	1.16E-38
Disease	1.13E-30	**Innate Immune System**	**3.81E-33**
Diseases of signal transduction by growth factor receptors and second messengers	1.16E-29	**Cytokine Signaling in Immune system**	**4.35E-33**
**Innate Immune System**	**7.67E-29**	Disease	1.51E-31
**MAPK family signaling cascades**	**2.49E-26**	Signaling by Interleukins	1.88E-31
Signaling by Interleukins	7.62E-25	Diseases of signal transduction by growth factor receptors and second messengers	1.13E-28
**Cytokine Signaling in Immune system**	**1.46E-24**	Intracellular signaling by second messengers	2.31E-26
Signaling by NTRK1 (TRKA)	3.94E-22	Toll-like Receptor Cascades	2.59E-24
Signaling by NTRKs	9.28E-22	**MAPK family signaling cascades**	**8.44E-24**
Toll-like Receptor Cascades	1.41E-21	**PI3K/AKT Signaling in Cancer**	**3.70E-20**
Intracellular signaling by second messengers	2.39E-21	Signaling by VEGF	4.27E-20
RAF/MAP kinase cascade	7.47E-20	VEGFA-VEGFR2 Pathway	6.27E-20
VEGFA-VEGFR2 Pathway	3.95E-19	**PIP3 activates AKT signaling**	**8.27E-20**
Toll Like Receptor 7/8 (TLR7/8) Cascade	1.70E-18	Toll Like Receptor 4 (TLR4) Cascade	2.11E-18
**PI3K/AKT Signaling in Cancer**	**1.74E-18**	Signaling by NTRK1 (TRKA)	2.54E-18
MyD88:MAL (TIRAP) cascade initiated on plasma membrane	2.71E-18	**MAPK1/MAPK3 signaling**	**3.35E-18**
Toll Like Receptor 4 (TLR4) Cascade	4.39E-18	MyD88:MAL (TIRAP) cascade initiated on plasma membrane	3.58E-18
MyD88 cascade initiated on plasma membrane	4.39E-18	Signaling by NTRKs	6.35E-18
**TRAF6 mediated induction of NFκB and MAP kinases upon TLR7/8 or 9 activation**	**1.09E-17**	Toll Like Receptor 7/8 (TLR7/8) Cascade	1.75E-17
Fc epsilon receptor (FCERI) signaling	6.63E-17	Axon guidance	2.82E-17
**PIP3 activates AKT signaling**	**7.82E-17**	Toll Like Receptor 9 (TLR9) Cascade	3.14E-17
**MAP kinase activation**	**1.28E-16**	Nervous system development	3.14E-17
Toll Like Receptor 3 (TLR3) Cascade	2.53E-16	MyD88 cascade initiated on plasma membrane	3.74E-17
Axon guidance	4.99E-16	RAF/MAP kinase cascade	4.81E-17
TRIF(TICAM1)-mediated TLR4 signaling	5.08E-16	**Negative regulation of the PI3K/AKT network**	**8.82E-17**
**Negative regulation of the PI3K/AKT network**	**7.08E-15**	**TRAF6 mediated induction of NFκB and MAP kinases upon TLR7/8 or 9 activation**	**9.82E-17**
**PI5P, *p*P2A and IER3 Regulate PI3K/AKT Signaling**	**2.87E-14**	Toll Like Receptor 3 (TLR3) Cascade	2.02E-15
**FCERI mediated MAPK activation**	**1.16E-12**	Fcgamma receptor (FCGR) dependent phagocytosis	7.91E-15

#### 3.1.3 Site-specific functional analysis

HIF-1α, a subunit of a transcription factor that promotes the expression of genes related to energy metabolism in response to hypoxic and normal oxygen conditions (Lu et al., 2002) is downstream of multiple pathways represented in Table 2 including, but not limited to Insulin signaling, PI3K-Akt, Mechanistic target of rapamycin (mTOR) signaling, MAPK signaling, as well as metabolic signaling, glycolysis and the citric acid cycle. Phosphorylation of T796 (query sites referred to in text, see tables for chicken site) is an activating phosphorylation (Gradin et al., 2002), and in the current study is phosphorylated in the ileum but not significantly altered in the jejunum. Casein kinase 2α (CK2α) is an enzyme which phosphorylates HIF-1α at T796, activating HIF-1α, and is significantly phosphorylated at T360 (resulting in increased CK2α activity) in ileum but is not significantly altered in jejunum. Additionally, S247, an inhibitory site on HIF-1α (Kalousi et al., 2010) shows a significant decrease in phosphorylation in the ileum but is not significantly altered in the jejunum. The phosphorylation of HIF-1α and its related proteins are summarized in Table 4.

**TABLE 4:** HIF-1α and related proteins’ phosphorylation events. Here, the kinome peptide array results are shown. Jejunum 500 and ileum 500 were compared to their respective control tissues. Phosphorylation events represented in this table show significant (*p*-value <0.05) for each site in ileum while these sites are not significantly altered in jejunum. Significant values are highlighted in bold.

UniProt ID	Protein	Query site	Chicken site	Peptide AA sequence	Jejunum 500 v Control		Ileum 500 v Control	
					Fold-change	*p*-value	Fold-change	*p*-value
Q16665	HIF-1α	T796	T781	ESGLPQL**T**SYDCEVN	1.078	0.165	**1.220**	**0.000**
Q16665	HIF-1α	S247	S247	KTFLSRH**S**LDMKFSY	-1.124	0.208	**-2.187**	**0.000**
P68400	CK2α	T360	T360	SGISSVP**T**PSPLGPL	1.082	0.105	**1.496**	**0.000**
Q15118	PDK1	Y243	Y215	AKSLCDL**Y**YMSSPEL	**-1.533**	**0.000**	**-1.493**	**0.003**
O15530	PDK1	S241	S245	SRQARAN**S**FVGTAQY	**1.194**	**0.013**	**1.172**	**0.010**

As HIF-1α activates glycolysis, it is also related to the uptake of glucose to feed this metabolic pathway (LaMonte et al., 2013). HIF-1α induces the expression of genes shown to be involved in the transport and metabolism of glucose (Harris, 2002; Semenza, 2009) while limiting the entry of metabolites into the mitochondria by activating pyruvate dehydrogenase 1 (PDK1) (Kim et al., 2006; Papandreou et al., 2006). PDK1’s activating site Y243 shows statistically significantly decreased phosphorylation in both ileum and jejunum.

The insulin signaling pathway incorporates the response to insulin (i.e., uptake of glucose) as well as glycolysis, MAPK, and PI3K-Atk (Kanehisa et al., 2021). A significant part of the insulin signaling pathway is PI3K-Akt signaling (along with MAPK and glycolysis). AKT Serine/Threonine Kinase 3 (AKT3), a central part of the PI3K-AKT signaling, shows altered phosphorylation only in the ileum. AKT3 shows decreased phosphorylation at S472/S476 (Sarbassov et al., 2005). AKT3 at site T305 is significantly increased in ileum and is not significant in jejunum. AKT3 and related peptide’s kinome data is summarized in Table 5.

**TABLE 5:** AKT3 and related proteins’ phosphorylation events. Here, the kinome peptide array results are shown. Jejunum 500 and ileum 500 were compared to their respective control tissues. Phosphorylation events represented in this table show significant (*p*-value <0.05) for sites as described above. Significant values are highlighted in bold.

UniProt ID	Protein	Query site	Chicken site	Peptide AA sequence	Jejunum 500 v Control		Ileum 500 v Control	
					Fold-change	*p*-value	Fold-change	*p*-value
Q9Y243	AKT3	S476	S476/S472	RPHFPQF**S**Y**S**ASGRE	-1.038	0.255	**-1.118**	**0.018**
Q9Y243	AKT3	T305	T305	TDAATMK**T**FCGTPEY	-1.075	0.126	**1.207**	**0.002**

mTOR is a serine/threonine kinase that affects a swath of cellular processes including cell growth, differentiation, and metabolism. mTOR induces transcription of HIF-1α and also regulates insulin signaling by altering Insulin Receptor Substrate 1 (IRS1), an early molecule in the insulin signaling pathway. IRS1 phosphorylation is increased in both tissues. mTOR phosphorylation was significantly altered in both ileum and jejunum as a result of feeding the product (Table 6). mTOR phosphorylates Eukaryotic translation initiation factor 4 E (eIF4E)-binding protein 1 (4EBP1) inducing transcription (Cheng et al., 2004). In the current study, 4EBP1 had significantly increased phosphorylation at S65 and T46 in both tissues, whereas, T37 had significantly increased phosphorylation in ileum but not in the jejunum. Regulatory-Associated Protein of mTOR Complex 1 (RPTOR) has two key sites for activation (S863 and T706). Both activation sites were statistically increased in ileum and unchanged in the jejunum.

**TABLE 6:** mTOR and related proteins’ phosphorylation events. Here, the kinome peptide array results are shown. Jejunum 500 and ileum 500 were compared to their respective control tissues. Phosphorylation events represented in this table show significant (*p*-value <0.05) for sites as described above. Significant values are highlighted in bold.

UniProt ID	Protein	Query site	Chicken site	Peptide AA sequence	Jejunum 500 v Control		Ileum 500 v Control	
					Fold-change	*p*-value	Fold-change	*p*-value
P42345	MTOR	S2448	S2352	RSRTRTD**S**YSASQSV	1.049	0.247	**1.231**	**0.002**
P42345	MTOR	S2481	S2387	TVPESIH**S**FIGDGLV	**1.269**	**0.000**	**-1.318**	**0.000**
Q8N122	RPTOR	S863	S864	LTQSAPA**S**PTNKGMH	1.111	0.062	**1.401**	**0.000**
P42345	MTOR	T2446	T2350	NKRSRTR**T**DSYSASQ	1.063	0.212	**1.179**	**0.008**
Q8N122	RPTOR	T706	T707	PAEGGSL**T**PVRDGPC	1.091	0.075	**1.323**	**0.000**
Q13541	EIF4EBP1	S65	S66	FLMECRN**S**PVAKTPP	**1.213**	**0.004**	**1.265**	**0.000**
Q13541	EIF4EBP1	T46	T47	GGTVFGT**T**PGGTRII	**1.228**	**0.001**	**1.248**	**0.000**
Q13541	EIF4EBP1	T37	T38	PPGDYST**T**PGGTVFG	-1.035	0.267	**1.165**	**0.007**

Numerous sites were significantly different between the tissues (Figure 1). mTOR’s phosphorylation site S2448 increased phosphorylation in ileum while no significant change is seen in jejunum. Increased phosphorylation also occurred in the ileum at site T2446, while no significant change was observed in jejunum. Further, site S2481 had significantly decreased phosphorylation in ileum.

The multiprotein complex formed by interaction of RPTOR and mTOR is called mTORC1. This complex has been shown to be essential for the phosphorylation of 4EBP1 (Hara et al., 2002).

Table 7 lists three 5’ AMP-activated protein kinase (AMPK) related phosphorylation sites within the kinome data. AMPK is an energy sensor that plays a role in cellular energy homeostasis (Hardie et al., 2012). Calcium/Calmodulin Dependent Protein Kinase 2 (CAMKK2) phosphorylates AMPK at S172 (activating site). CAMKK2 phosphorylation (S511) was significantly increased in ileum, but not jejunum. AMPK beta-2 at site S108 is shown to be an activating phosphorylation (Warden et al., 2001), and in the current study showed statistically significantly decreased phosphorylation in both the ileum and jejunum.

**TABLE 7:** AMPK and related proteins’ phosphorylation events. Here, the kinome peptide array results are shown. Jejunum 500 and ileum 500 were compared to their respective control tissues. Phosphorylation events represented in this table show significant (*p*-value <0.05) for sites as described above. Significant values are highlighted in bold.

UniProt ID	Protein	Query site	Chicken site	Peptide AA sequence	Jejunum 500 v Control		Ileum 500 v Control	
					Fold-change	*p*-value	Fold-change	*p*-value
Q13131	AMPK (PRKAA1)	S172	S174	KIADFGL**S**NMMSDGE	-1.066	0.142	**1.193**	**0.004**
O43741	AMPK (PRKAB2)	S108	S110	TKIPLIK**S**HNDFVAI	**-1.299**	**0.001**	**-1.442**	**0.000**
Q96RR4	CAMKK2	S511	S483	RREERSL**S**APGNLLP	1.084	0.098	**1.145**	**0.008**

As described above, PI3K-Atk signaling is part of insulin signaling. Akt activates NFκB by phosphorylating Inhibitor of nuclear factor kappa-B kinase subunit alpha (IKKa) (Nidai Ozes et al., 1999; Romashkova and Makarov, 1999). NF-kB transcription factor activity is crucial in immune and inflammatory processes, cell cycle and proliferative processes, and cell death. Phosphorylation of NFκB is summarized in Table 8. NF-kB p50 (S337) shows increased phosphorylation in the ileum. This site is important for DNA binding (Hou et al., 2003; Guan et al., 2005). NF-kB p105 (S932) shows increased phosphorylation in the jejunum. This site has been shown to allow for the processing for p105 to p50 (active form of subunit) (Heissmeyer et al., 1999; Lang et al., 2003). IKKa (T23) phosphorylation activates the kinase, which then phosphorylates nuclear factor of kappa light polypeptide gene enhancer in B-cells inhibitor, alpha (IkBa) (inhibitor of NFκB) which then dissociates from NFκB allowing NFκB to translocate to the nucleus (Cahill and Rogers, 2008). IKKa (T23) is phosphorylated in ileum, not significant in jejunum. IKKa (S176/S180, S180 is on the array, S176 is included in the peptide) both activating phosphorylations (Ling et al., 1998; Senftleben et al., 2001). This site shows increased phosphorylation in jejunum while not significantly altered in ileum. Inducible IκB kinase (IKK-i) autophosphorylates at S172, this is an activating phosphorylation which results in activation of activates NFκB. (Shimada et al., 1999). IKK-i shows increased phosphorylation in Ileum while not being significant in jejunum. COX-2 is activated by phosphorylation at Y446 (Alexanian et al., 2014). This site is significantly increased in the ileum and not significantly altered in the jejunum. NFκB is reported to induce HIF-1α *via* a multitude of cellular stimuli including Interleukin-1beta (IL-1β) and Tumour Necrosis Factor alpha (TNFα) mediated signaling.

**TABLE 8:** NFκB and Related proteins’ phosphorylation events. Here, the kinome peptide array results are shown. Jejunum 500 and ileum 500 were compared to their respective control tissues. Phosphorylation events represented in this table show significant (*p*-value <0.05) for sites as described above. Significant values are highlighted in bold.

UniProt ID	Protein	Query site	Chicken site	Peptide AA sequence	Jejunum 500 v Control		Ileum 500 v Control	
					Fold-change	*p*-value	Fold-change	*p*-value
P19838	NFΚB1	S337	S345	FVQLRRK**S**DLETSEP	-1.038	0.2426	**1.080**	**0.038**
P19838	NFΚB1	S932	S946	CDSGVET**S**FRKLSFT	**1.115**	**0.028**	1.106	0.064
O15111	CHUK	T23	T37	EMRDRLG**T**GGFGNVC	-1.058	0.124	**1.239**	**0.000**
O15111	CHUK	S180/S176	S194	DQG**S**LCT**S**FVGTLQY	**1.310**	**0.000**	1.097	0.130
Q14164	IKBKE	S172	S172	EDDEKFV**S**VYGTEEY	1.055	0.120	**1.141**	**0.012**
Q05769	COX2	Y446	Y446	DQSRQMR**Y**QSLNEYR	1.014	0.412	**1.226**	**0.001**

### 3.2 Quantitative RT-PCR to measure mRNA expression

**FIGURE 3:**
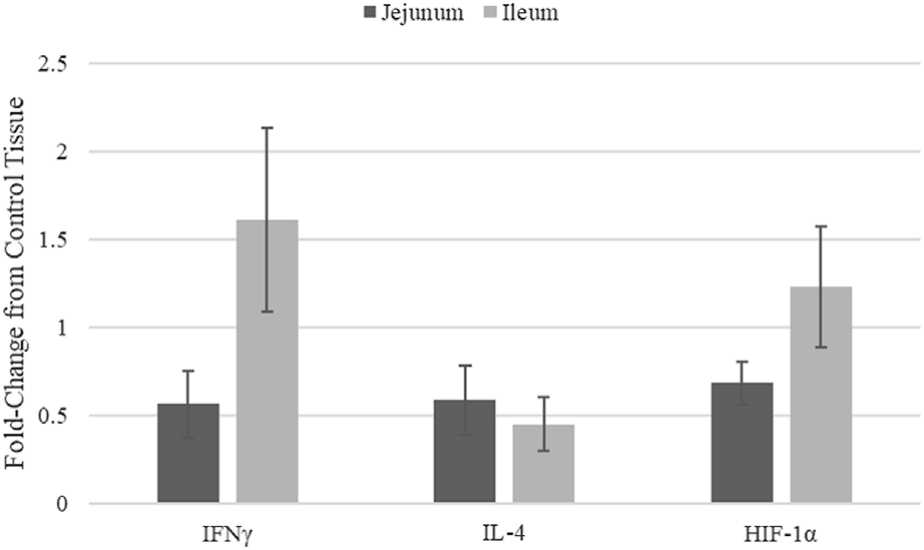
mRNA Expression in jejunum and ileum samples from blend-fed chickens compared to those on control diet.

Quantitative real-time RT-PCR was used to validate kinome peptide array data analysis (Figure 3). ThemRNA expression levels of interferon gamma (INFγ). Interleukin 4 (IL-4), and HIF-1α in jejunum and ileum were measured. The mRNA expression was compared in tissues from broilers fed the microencapsulated bend relative to their respective control tissues. Fold-change (FC) was calculated using the 40^−ΔΔCT^ method and error bars show Standard Error. FC for IFNγ, IL-4, and HIF-1α in jejunum were 0.57, 0.59, and 0.69 respectively. FC for IFNγ, IL-4, and HIF-1α in Ileum were 1.61, 0.45, and 1.23 respectively. Student’s *t*-test was performed and *p*-values for IFNγ, IL-4, and HIF-1α for jejunum were 0.12, 0.57, 0.15 respectively, while in ileum *p*-values were 0.78, 0.09, and 0.92 respectively.

Though mRNA expression is not necessarily correlated with protein translation or protein activity, coupling gene expression data with the kinome data provides a method of validating the signal transduction observed in the kinome data. Key indicators of immune stimulation (IFNy (Figure 3), other immune genes from (Swaggerty et al., 2020a)) and glycolysis related genes (HIF-1α) show increases in the ileum vs. the jejunum, in agreement with the kinome data. HIF-1α transcription does not necessarily mean HIF-1α function is active, however this in conjunction with Kinome data indicate increased activity in ileal tissue, suggest that HIF-1α is indeed functioning.

### 3.3 Summary of results

**FIGURE 4:**
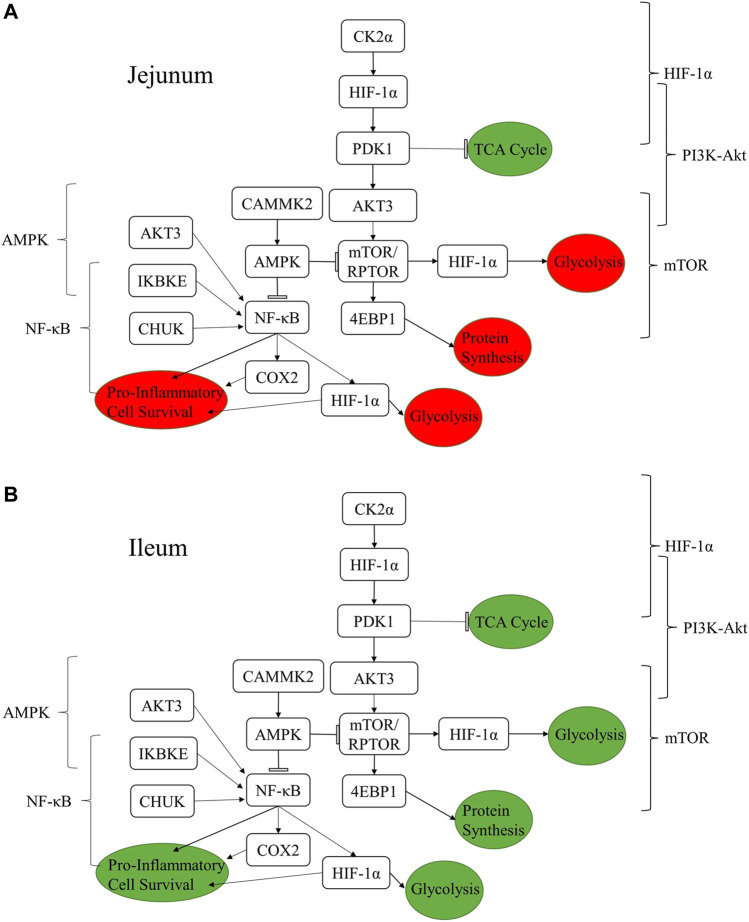
Summary figure showing the relationships between proteins discussed in the results and the resulting functional changes imparted by the product in jejunum **(A)** and ileum **(B)**. The brackets on either side of the figure denote the relevant signaling pathway. The colored circles represent the downstream functional effects of the signaling represented. The colors of the circles show whether the downstream functional effect is increased (green) or decreased (red).

Figure 4 summarizes how the proteins listed in Tables 4– 8 are related to one another and how a selection of relevant pathways (Tables 2, 3) come together to form this network. The consequences of the signaling changes result in changes to biological and metabolic processes. In jejunum, the signaling changes observed using both the kinome data and qPCR results indicate an increase in tricarboxylic acid cycle (TCA cycle) signaling and a reduction in pro-inflammatory processes, cell survival, protein synthesis, and glycolysis. In ileum, the same increase in TCA cycle as seen in the jejunum is observed, however the other processes were also increased in the ileum, whereas they were decreased in the jejunum.

## 4 Discussion

The botanical blend product studied here has been shown previously to have both a performance and disease mitigation effects (EFSA Panel on Additives and Products or Substances used in Animal Feed (FEEDAP) et al., 2020; Swaggerty et al., 2020a; Swaggerty et al., 2022). Here we attempt to describe a mechanism of action behind those observations. A possible mechanism for the improved chicken growth and performance shown previously is the enhanced glycolysis related signaling observed in this study in the ileum. This enhanced glycolysis occurs in the presence of adequate oxygen and can be considered aerobic glycolysis, otherwise known as the Warburg effect (Vaupel and Multhoff, 2021). In both tissues you see altered glycolysis, in the ileum which results in signaling that may lead to the enhanced production of anabolic building blocks, in the jejunum the lack of glycolytic signaling resulted in decreased expression of cytokines and chemokines typically associated with inflammation (Tables 4– 8; Figure 3). Rapidly growing or highly active cells require a high level of metabolites. Many of these metabolites come in the form of glycolytic or TCA cycle intermediates. By increasing aerobic glycolysis, a ready pool of metabolites and NADPH reducing agents are available for growth and activity (Vander Heiden et al., 2009). The Warburg effect was first discovered in cancer, a situation of high proliferation. More recently this phenotype has been found in immune cells and rapidly developing tissue as well. A phenotype similar to a growing broiler chicken (Johnson, 2021).

Insulin signaling activates AKT which activates mTOR which induces the transcription factors HIF-1 and NFκB (DeBerardinis et al., 2008). HIF-1 induces pro-glycolysis genes such as hexokinase, among others, which activates and perpetuates the cycle of increased glycolysis leading to greater HIF-1 activity (Semenza, 2013; Cheng et al., 2014). Pyruvate dehydrogenase kinase 1’s (PDK1) activating site Y243 shows statistically significantly decreased phosphorylation in both ileum and jejunum. This site being phosphorylated is shown to increase PDK1’s activity and increase conversion of pyruvate into lactate (Hitosugi et al., 2011), supporting a pro-glycolysis/Warburg phenotype in the ileum and a reduction in said signaling in jejunum. NFκB is activated *via* insulin signaling/Akt which induces the expression of HIF-1α and several pro-inflammatory cytokines (several of which show increased expression in the ileum and not in the jejunum) as seen published previously with this product (Swaggerty et al., 2020a; Swaggerty et al., 2022). These results describe the mechanistic pathway leading to the increased glycolysis in the ileum as well as the mRNA expression results observed previously. This was accomplished by tracing the activation status and upstream signaling events leading to the activation of NFκB, which is a transcription factor transcribing proinflammatory cytokines and chemokines (Swaggerty et al., 2020a).

Previous research has shown that this product enhances growth and improves outcomes related to necrotic enteritis (NE) (Swaggerty et al., 2022). qPCR in that previous study (Swaggerty et al., 2022), looking at the same blend in the context of a NE model, mRNA expression in the jejunum for interleukin 6 (IL-6), interleukin 10 (IL-10), and IFNy were all increased (10.9, 5.03, and 5.66-fold respectively). In the context of this study, without a disease challenge, the blend resulted in reduced mRNA expression of IL-6 and IFNy (0.57 and 0.6 respectively) and less of an increase in IL-10 (1.39). These data support the hypothesis that the blend results in a more quiescent (potentiated) state in the jejunum and increases the capacity to respond to challenge. The inflammatory response to a pathogen is critical to the clearance of the pathogen. Jejunum is a more important site of nutrient absorption and also a more basally inflammatory tissue than ileum (Johnson et al., 2020). This may be explained by the fact that immune cells are more represented in the jejunum than the ileum, thus we observe changes that may represent a more growth effect in the ileum and anti-inflammatory effect in the jejunum.

Anti-inflammatory effects seen in the jejunum may be allowing for better barrier integrity and function, while allowing more proliferative signaling systemically (as reflected in ileum) thus better chicken growth with less permeable (leaky gut) GI tract. Thus, feed additive’s primary function appears to be to decease inherent innate immune signaling, at least in the gut. This interpretation of our results is supported by the findings in a recent publication by Bialkowski et al. (2023) which showed an increase in gut barrier function in broilers in response to the same product reported on here. This increases the capacity to respond in the event of a disease or stress challenge (Perry et al., 2022; Swaggerty et al., 2022). You see more of an increase in cytokine expression and pro-inflammatory signaling in the jejunum with the treatment fed groups in the context of a challenge, but as reported here, in the absence of a challenge the gut is relegated to a more quiescent (potentiated) state. This means that the products are enabling greater capacity, or potential, to respond to these challenges. Given the results presented here, as well as the results referenced from studies testing similar products, it would be prudent to conduct a study that looks not only at the effect of this or similar products on the gut tissues, but more systematically as well. Previous work has shown that a subclinical *Salmonella* challenge can have a measurable effect on muscle metabolism (Kogut et al., 2016). As such, a specific site of further study should be the muscle tissue, as a major site of nutrient utilization in a broiler chicken it would be very likely to show effects related to any systemic change in proliferative and/or inflammatory signaling.

The jejunum and ileum are both segments of the small intestine and are being exposed to the same product. These segments have distinct physiological functions related to nutrient absorption and immunity (Svihus, 2014). The kinome data shows a clear distinction in the activation status of key immunometabolic signaling pathways (Tables 2, 3) between jejunum and ileum following the feeding of the blend product. These pathways are active in the ileum and absent or turned off in the jejunum. While some have argued that there is no anatomical or physiological distinction to be made between the jejunum and ileum in a chicken (Duke, 1986) our results here and elsewhere (Swaggerty et al., 2020a) have shown significant differences along the GI tract, not only between duodenum, cecum and ileum/jejunum but stark differences between the ileum and jejunum as well (Bialkowski et al., 2023). This post-translational and gene expression differences highlight the need for researchers to study multiple segments of the intestine in order to generate a clear picture of challenge or intervention effects.

Though many of the same pathways were altered by the product, as measured by phosphorylation changes in the proteins of those pathways, in both the jejunum and ileum, the members of the pathways show differential responses between the tissues as reflected in the changes in phosphorylation status of individual phosphorylation sites (Figures 1, 2). These differences show how in one tissue (Jejunum) proliferative signaling (transcription factors, gene expression) can be curtailed while in an adjacent tissue (Ileum) proliferative signaling (transcription factors, gene expression) can be heightened in response to the same treatment. Though, it is possible part of this difference is due to different concentrations of the product as it passes along the GI tract (Grilli et al., 2007; Piva et al., 2007). Future studies could evaluate the differential effects between these gut segments by utilizing organoids designed to represent the ileum and jejunum. Research is currently being done to develop and utilize organoids for such studies, however the technology may not yet be advanced enough to represent different segments of the gut perfectly (Li et al., 2018; Ghiselli et al., 2021; Nash et al., 2021; Zhao et al., 2022). Looking at the site-specific functions of phosphorylation status allows for determining the mechanism of action of the product in a context specific way (Tables 4– 8). These site-specific changes highlight the importance of determining the functional effect of differential phosphorylation rather than rely on the phosphorylation status itself (increased phosphorylation doesn’t always mean on nor the inverse).

## Data Availability

The raw data supporting the conclusion of this article will be made available by the authors, without undue reservation.
